# Association between metformin use prior to admission and lower mortality in septic adult patients with diabetes mellitus: beware of potential confounders

**DOI:** 10.1186/s13054-020-02909-3

**Published:** 2020-04-28

**Authors:** Patrick M. Honore, Aude Mugisha, Luc Kugener, Sebastien Redant, Rachid Attou, Andrea Gallerani, David De Bels

**Affiliations:** ICU Department, Centre Hospitalier Universitaire Brugmann-Brugmann University Hospital, Place Van Gehuchtenplein, 4, 1020 Brussels, Belgium

We read with great interest the recent paper by Liang et al. who conclude that their meta-analysis indicated an association between metformin (MET) use prior to admission and lower mortality in septic adult patients with diabetes mellitus [1]. We would like to make some comments. Nearly half of critically ill patients, especially those with septic shock, have or develop acute kidney injury (AKI), and 20–25% need renal replacement therapy (RRT) within the first week of their admission [2]. Because of its low molecular weight and minimal protein binding, metformin is equally (highly) eliminated by ultrafiltration (convection) and dialysis (diffusion). Furthermore, its large volume of distribution within a two-compartment pharmacokinetic model implies that metformin may be more effectively cleared by prolonged RRT. This was corroborated by Keller et al., who showed a dramatic reduction of metabolic acidosis and metformin plasma concentrations within the first 24 h after initiating CRRT in patients with MET-induced lactic acidosis, followed by normalization on the second day in all subjects [3]. Although we do not know the exact rate of CRRT in both arms [1], it may well be that one group had more CRRT than the other, particularly the metformin group. For instance, in the study of Doenyas-Barak et al., which had a huge impact on the conclusions of this meta-analysis, the use of RRT was higher in the MET-treated population (38.6 vs. 21.2%, *p* = 0.13) [1, 4]. Accordingly, we suspect that the observed difference in mortality rate may be due to the more frequent use of RRT in the MET-treated population. A protective effect of RRT has already been suggested by Peters et al., who found that despite higher illness severity, the mortality rate in patients with MET-associated lactic acidosis treated with intermittent hemodialysis (IHD) was no different to that of non-dialyzed subjects [5].

## Data Availability

Not applicable.

## Abbreviations

MET: Metformin
AKI: Acute kidney injury
RRT: Renal replacement therapy
IHD: Intermittent hemodialysis
CRRT: Continuous renal replacement therapy
